# Transforming Ophthalmology Training via Mobile Learning during the COVID-19 Pandemic

**DOI:** 10.18502/jovr.v16i4.9762

**Published:** 2021-10-25

**Authors:** Mohammad-Mehdi Sadoughi, Soleiman Ahmady, Masomeh Kalantarion, Nasrin Khajeali

**Affiliations:** ^1^Ophthalmic Research Center, Research Institute for Ophthalmology and Vision Science, Shahid Beheshti University of Medical Sciences, Tehran, Iran; ^2^Department of Medical Education, Virtual School of Medical Education and Management, Shahid Beheshti University of Medical Sciences, Tehran, Iran; ^3^Department of Medical Education, Ahvaz Jundishapur University of Medical Sciences, Ahvaz, Iran

##  Dear Editor,

The coronavirus disease 2019 (COVID-19) pandemic was reported as a public health crisis. The quick spread of COVID-19 has had a huge effect on many aspects of our life, including social development and education.^[1]^ It has forced measures of social distancing and challenges in the delivery of “in-person” education. Medical universities, involved in training ophthalmologists, are using innovative strategies to continue their standard of education.^[2,3]^

In this letter, we discuss a new strategy that was utilized to maintain ophthalmic education during this time of the COVID-19 pandemic. One of the pioneering strategies that played a vital role during this pandemic is mobile learning. Mobile learning is a learning strategy that supports the mobility of students as it facilitates learning at any time and place via the use of mobile devices.^[4]^

The advantages and disadvantages of mobile learning has been widely discussed by many researchers.^[5,6]^ Mobile learning is helping to promote knowledge and learning beyond the walls of the classroom and also facilitate continued student–teacher relations. Other inherent advantages of mobile learning are that it is ubiquitous, portable, hybrid, interactive, and coordinative.

On the other hand, some of the inescapable disadvantages of mobile learning include the use of small screens, the loss of security, the low knowledge of mobiquette, and the experience of virtual fatigue. However, advances in technology have provided alternatives to the use of small screens and have also enhanced the security of using these devices. It was also discovered that interactive webinar mobile applications (apps) might decrease the experience of virtual fatigue.^[4]^

Mobile learning has afforded us the opportunity to not only improve our knowledge but also improve our skills through simulation, which is very useful in ophthalmology education. During their period of training, residents should execute qualifying technical ophthalmic surgeries. As a result of social distancing, patients prefer to stay at home instead of being referred to educational hospitals for ophthalmic visits. Consequently, ophthalmology residents have had a significant reduction in their surgical activities. To respond to this challenge, certain educational mobile platforms were recommended. One such recommendation was “Touch Surgery”, which is a surgical simulation app that includes ophthalmology and 11 other surgical specialties. These surgical simulation apps increase procedural proficiency and lessen intraoperative errors.^[5]^

In this crisis, social media has also played a vital role in ophthalmic education. One such social media platform is Twitter, which provides a forum for connecting peer students with ophthalmology educators who can address the educational needs of their students.

Mobile learning has also facilitated the use of innovative and interactive smartphone apps that are suitable learning resources for ophthalmologists. These applications that have been developed such as American Academy of Ophthalmology Ophthalmic Education App, Ophthalmology Guide app, Ophthalmic Instruments app, Eye Handbook app,^[7]^ can be utilized for a range of topics, from teaching the anatomy via virtual reality or augmented reality to learning clinical information, self-assessment, and procedural skills.

In summary, we have discovered that through mobile learning, the utilization of these innovative applications may expand opportunities for accessing standard ophthalmic education while also facilitating student-to-student and student-to-teacher interactions. During the COVID-19 period, mobile learning can be a valid solution to the ophthalmology-training gap experienced because of the social restrictions.

##  Financial Support and Sponsorship

Nil.

##  Conflicts of Interest

There are no conflicts of interest.
